# Parasitosis caused by *Calyptospora* in *Pygocentrus nattereri* (Characiformes: Serrasalmidae) from the Sacaizal Lake in the state of Amapá, Brazil

**DOI:** 10.1590/S1984-29612025070

**Published:** 2025-12-08

**Authors:** Nayana Moraes de Sena, Jhonata Eduard, Camila Maria Barbosa Pereira, Maria do Perpetuo Socorro Progene Vilhena, José Francisco Berrêdo Reis da Silva, José Ledamir Sindeaux, Michele Velasco

**Affiliations:** 1 Universidade Federal Rural da Amazônia – UFRA, Instituto de Saúde e Produção Animal – ISPA, Laboratório e Tecnologias de Integração Morfo-Molecular – LIMT, Belém, PA, Brasil; 2 Universidade Federal Rural da Amazônia – UFRA, Programa de Pós-Graduação em Saúde e Produção Animal na Amazônia – PPGSPAA, Belém, PA, Brasil; 3 Universidade Federal do Pará – UFPA, Programa de Pós-Graduação em Biologia de Agentes Infecciosos e Parasitários – BAIP, Belém, PA, Brasil; 4 Universidade Federal do Pará – UFPA, Programa de Pós-Graduação em Biodiversidade e Biotecnologia – PPG-BIONORTE, Belém, PA, Brasil; 5 Universidade Federal Rural da Amazônia – UFRA, Centro de Tecnologia Agropecuária – CTA, Belém, PA, Brasil; 6 Museu do Pará Emílio Goeldi – MPEG, Belém, PA, Brasil; 7 Universidade Federal Rural da Amazônia – UFRA, Pós-Graduação em Reprodução Animal na Amazônia – REPROAMAZON, Belém, PA, Brasil

**Keywords:** Coccidiosis, fish, Brazil, histopathology, fibrosis, Coccidiose, peixes, Brasil, histopatologia, fibrose

## Abstract

Parasites of the phylum Apicomplexa are among the main etiological agents of diseases in vertebrates and invertebrates, including humans and domestic animals. In the present study, 18 specimens of *Pygocentrus nattereri* (red piranha) captured in Lagoa do Sacaizal, municipality of Pracuúba, state of Amapá, were analyzed. Of these, 6 (33%) had whitish spots on their liver lobes. Clusters of *Calyptospora* sp. oocysts measuring 18.1 ± 0.5 (17.4-18.7) µm in diameter, with four pyriform sporocysts measuring 9.0 ± 0.45 (8.3-9.8) µm in length and 4.3 ± 0.50 (3.7-5.1) µm in width, each with two sporozoites. Histopathological evaluation indicated the presence of oocysts adjacent to blood vessels and in the hepatopancreas, promoting necrosis, degeneration, and tissue fibrosis associated with inflammatory infiltrate and melanomacrophage bodies. This is the first record of coccidia parasitizing *P. nattereri*.

## Resumo

Parasitos do filo Apicomplexa estão entre os principais agentes etiológicos de doenças em vertebrados e invertebrados, incluindo humanos e animais domésticos. No presente estudo, foram analisados 18 espécimes de *Pygocentrus nattereri* (piranha vermelha) capturados na Lagoa do Sacaizal, município de Pracuúba, estado do Amapá. Destes, 6 (33%) apresentaram manchas esbranquiçadas nos lobos hepáticos. Foram observados aglomerados de oocistos de *Calyptospora* sp. medindo 18,1 ± 0,5 (17,4-18,7) µm de diâmetro, com quatro esporocistos piriformes medindo 9,0 ± 0,45 (8,3-9,8) µm de comprimento e 4,3 ± 0,50 (3,7-5,1) µm de largura e cada um com dois esporozoítos. A avaliação histopatológica indicou a presença dos oocistos adjacentes aos vasos sanguíneos e no hepatopâncreas, promovendo necrose, degeneração e fibrose tecidual associada a infiltrado inflamatório e corpos melanomacrófagos. Este é o primeiro registro da ocorrência de coccídio parasitando *P. nattereri*.

*Pygocentrus nattereri* Kner, 1858*,* popularly known as "red piranha", is a serrasalmid belonging to the most diverse group of fish, the Characiformes, with valid species of importance for regional fishing and aquaculture. The species has a wide distribution and is present in several river basins throughout South America (Nelson et al., 2016).

Organisms of the phylum Apicomplexa Levine, 1970, are among the most important etiological agents responsible for diseases in vertebrates and invertebrates, including humans and domestic animals, with significant environmental, economic, and public health implications (Silva et al., 2012). They possess an apical complex composed of specialized secretory organelles and produce resistant cysts that enable direct transmission through the food chain between predators and prey or through arthropod vectors (Votýpka et al., 2016).

The class Coccidia Leuckart, 1879 comprises a diverse group of protozoa with a parasitic lifestyle, affecting natural stocks of several fish species, with important characteristics regarding parasitic invasion and transmission ecology. *Calyptospora* Overstreet, Hawkins & Fournie, 1984, preferentially infects the hepatic region of fish. Its morphological characteristics include oocysts with four sporocysts, each containing two sporozoites (Fournie et al., 1985). The sporocysts can vary from round to ellipsoid in morphology.

Currently, eight species of *Calyptospora* have been described as infecting fish. Of these species, three occur in marine fish: *C. funduli* (Duszynski, Solangi & Overstreet, 1979) in fishes of the family Fundulidae such as *Fundulus grandis* Baird & Girard, 1853, *F. heteroclitus* (Linnaeus, 1766), *F. jenkinsi* (Evermann, 1892), *F. pulvereus* (Evermann, 1892), *F. similis* (Baird & Girard, 1853) and *Menidia beryllina* (Cope, 1867) (Atherinidae) from the coastal area of ​​Mississippi (Fournie & Overstreet, 1993); *C. matosi* Neto, Székely, Eduard et al., 2025, identified in *Bagre bagre* (Linnaeus, 1766) (Ariidae) in Bragança (Ajuruteua beach) and Curuçá (São João do Abade district) in Pará state, Brazil (Araujo Neto et al., 2025); and *C. virescentis* Eduard, Azevedo, Pereira et al., 2025 in *Cynoscion virescens* (Cuvier, 1830) (Sciaenidae), a marine and estuarine fish from Curuçá, northeastern Pará, Brazilian coast, Brazil (Eduard et al., 2025). The greatest diversity of *Calyptospora* is found in freshwater fish, mainly in the Brazilian Amazon, according to Table 1.

**Table 1:** Freshwater hosts of *Calyptospora*. Measurements in micrometers (µm).

**Order of Fish**	**Host**	**Location**	**Parasite species**	**Site of infection**	**Oocyst diameter**	**Sporocysts**			**Reference**
						**Shape**	**Length**	**Width**	
**Characiformes**	*Pygocentrus nattereri* Kner, 1858 - Serrasalmidae	Lake Sacaizal, Pracuúba, Amapá, Brazil	*Calyptospora* sp.	Liver	18.1	Pyriform	9.0	4.3	**Present study**
	*Serrasalmus striolatus* (Steindachner, 1908) -Serrasalmidae, *S. rhombeus* (Linnaeus, 1766) - Serrasalmidae	Amazon River, Belém and lagoon region of the “Dois Irmãos” Zoo, Recife, Brazil	*Calyptospora serrasalmi*	Liver	25.5	Pyriform	11.8	6.0	Casal et al. (2007)
	*Triportheus guentheri* (Garman, 1890) - Triportheidae	Three Marias Reservoir, São Francisco River, Brazil	*Calyptospora* sp.	Liver	24.5	Ellipsoidal	11.5	4.5	de Albuquerque & de Carvalho Brasil-Sato (2010)
	*Tetragonopterus chalceus* Spix & Agassiz, 1829 - Characidae			Intestine					
	*Tetragonopterus chalceus* Spix & Agassiz, 1829 - Characidae	Three Marias Reservoir upper São Francisco, Minas Gerais, Brazil.	*Calyptospora* sp.	Liver	-	-	-	-	de Albuquerque et al. (2016)
	*Triportheus guentheri* (Garman, 1890) - Characidae								
	*Hoplias malabaricus* (Bloch, 1794) - Erythrinidae	Curiaú river, Macapá, Amapá, Brazil	*Calyptospora serrasalmi*	Liver	23.4	Pyriform	8.2	4.0	Silva Negrão et al. (2019)
	*Triportheus Angulatus* (Spix & Agassiz, 1829) -Triportheidae	Tocantins River,Maranhão, Brazil	*Calyptospora gonzaguensis*	Liver, Gallbladder, adipose tissue	19.6	Ellipsoidal	9.2	3.9	Silva et al. (2020)
	*Serrasalmus rhombeus* (Linnaeus, 1766) - Serrasalmidae	Grass River, Ipixuna of Pará, Pará, Brazil	*Calyptospora* sp.	Liver	17.4	Pyriform	8.3	4.3	Oliveira et al. (2021)
	*Hoplias malabaricus* (Bloch, 1794) - Erythrinidae	Três Marias reservoir, São Francisco river, Minas Gerais, Brazil	*Calyptospora* sp.	Liver	-	-	-	-	Duarte et al. (2023)
**Cicliformes**	*Cichla ocellaris* Bloch & Schneider, 1801 - Cichlidae	Reservoirs: Curfi and Jaguaribe River Basins - State of Ceará, Serido River Basin - State of Rio Grande do Norte, and Experimental Ichthyological Research Center Fish Farming, Pentecoste, Brazil	*Calyptospora tucunarensis*	Liver	24.3	Ellipsoidal	8.3	3.7	Békési & Molnár (1991)
	*Crenicichla lepidota* Heckel , 1840 - Cichlidae	Amazon River, Belém, Brazil	*Calyptospora spinosa*	Liver, testes and ovaries	22.3	Ellipsoidal	9.3	3.8	Azevedo et al. (1993)
	*Cichla temensis* Humboldt, 1821 - Cichlidae	Marajó-Açu River, Marajó Island, Pará, Brazil	*Calyptospora* sp.	Liver	21.2	Pyriform	9.2	3.1	Santiago et al. (2012)
	*Aequidens plagiozonatus* Kullander , 1984 - Cichlidae	Municipality of Peixe-Boi, Pará, Brazil	*Calyptospora* sp.	Hepatopancreas	-	-	-	-	Videira et al. (2013)
	*Cichla piquiti* Kullander &Ferreira, 2006 - Cichlidae	Estreito hydroelectric reservoir - Tocantins River, Maranhão, Tocantins, Brazil	*Calyptospora paranaidji*	Liver	22.1	Ellipsoidal	9.7	4.6	Silva et al. (2019)
	*Geophagus proximus* (Castelnau , 1855)	Curiaú river, Macapá, Amapá, Brazil	*Calyptospora serrasalmi*	Liver, gallbladder and heart	22.5	Pyriform	9.0	4.0	Silva Negrão et al. (2019)
	*Cichla monoculus* Agassiz , 1831 - Cichlidae	Lake Sacaizal, Pracuúba, Amapá, Brazil	*Calyptospora* sp.	Liver	21.0	Pyriform	8.7	4.9	Amoras et al. (2024)
	*Mayaheros urophthalmus* (Günther , 1862) - Cichlidae and *Parachromis friedrichsthalii* (Heckel , 1840) - Cichlidae	Three water sources (Baldiosera, Muuch and San Crisanto), Yucatán, México	*Calyptospora mexicanus*	Liver	25.3	Pyriform	13.7	5.4	Colunga-Ramírez et al. (2025)
**Siluriformes**	*Brachyplatystoma vaillantii* (Valenciennes , 1840) - Pimelodidae	Paracauari River, in Salvaterra, Marajó Island, Pará, Brazil	*Calyptospora* sp.	Liver	21.0	Pyriform	9.2	4.5	Silva et al. (2012)
	*Rhamdia guatemalensis* (Günther, 1864) - Heptapteridae	San Crisanto water spring, Yucatán, México	*Calyptospora mexicanus*	Liver and gallbladder	25.3	Pyriform	13.7	5.4	Colunga-Ramírez et al. (2025)
**Cyprinodontiformes**	*Fundulus notti* (Agassiz , 1854) - Fundulidae	Fourmile Branch of Moungers Creek, Jackson County, Mississippi, USA	*Calyptospora empristica*	Hepatocytes and pancreatic acinar cells	22.0	Spherical	9.0	5.0	Fournie et al. (1985)
**Osteoglossiformes**	*Arapaima gigas* (Schinz, 1822 ) - Arapaimidae	Manaus, Brazil	*Calyptospora* sp.	Liver	19.0	Pyriform	9.0	4.0	Bonar et al. (2006)

This study aimed to describe the morphological and histopathological changes caused by *Calyptospora* sp. in the liver of *P. nattereri* captured from Lake Sacaizal (1°42'8.79"N 50°43'17.56"W) in the municipality of Pracuúba, Amapá State, Brazil.

Eighteen specimens of *P. nattereri* (SISBIO/ICMBio License nº 88196-1) were acquired from artisanal fishermen, placed in isothermal boxes with ice, and transported to the Morpho-molecular Integration and Technologies Laboratory at the Federal Rural University of Amazônia in Belém, Pará, for necropsy (CEUA no. 7218270723 /ID000609). Subsequently, the entire body surface, tissues, and organs were analyzed using a stereoscopic microscope and a light microscope to identify for parasites and perform morphological analyses.

The methodology of Bush et al. (1997), was used to calculate prevalence of fish examined. In the morphometric analysis, the oocysts (n = 20) and their internal structures were measured in micrometers (µm) using ImageJ version 1.46r software, and mean and standard deviation values were subsequently calculated. The Parasitized liver tissue fragments were fixed in Davidson's solution for 24 h. Subsequently, the sections were dehydrated in an increasing series of ethanol, diaphanized in xylol, and embedded in paraffin. Sections of 5 μm thickness were obtained using a microtome and stained with hematoxylin & eosin and Gomori special trichrome stain.

For scanning electron microscopy, the samples were prepared according to Azevedo et al. (1995). Visualizations and photomicrographs were obtained using a Tescan Mira3 scanning electron microscope at the Scanning Electron Microscopy Laboratory of the Research Campus of the Museu Paraense Emílio Goeldi-MPEG.

**Figure 1:**
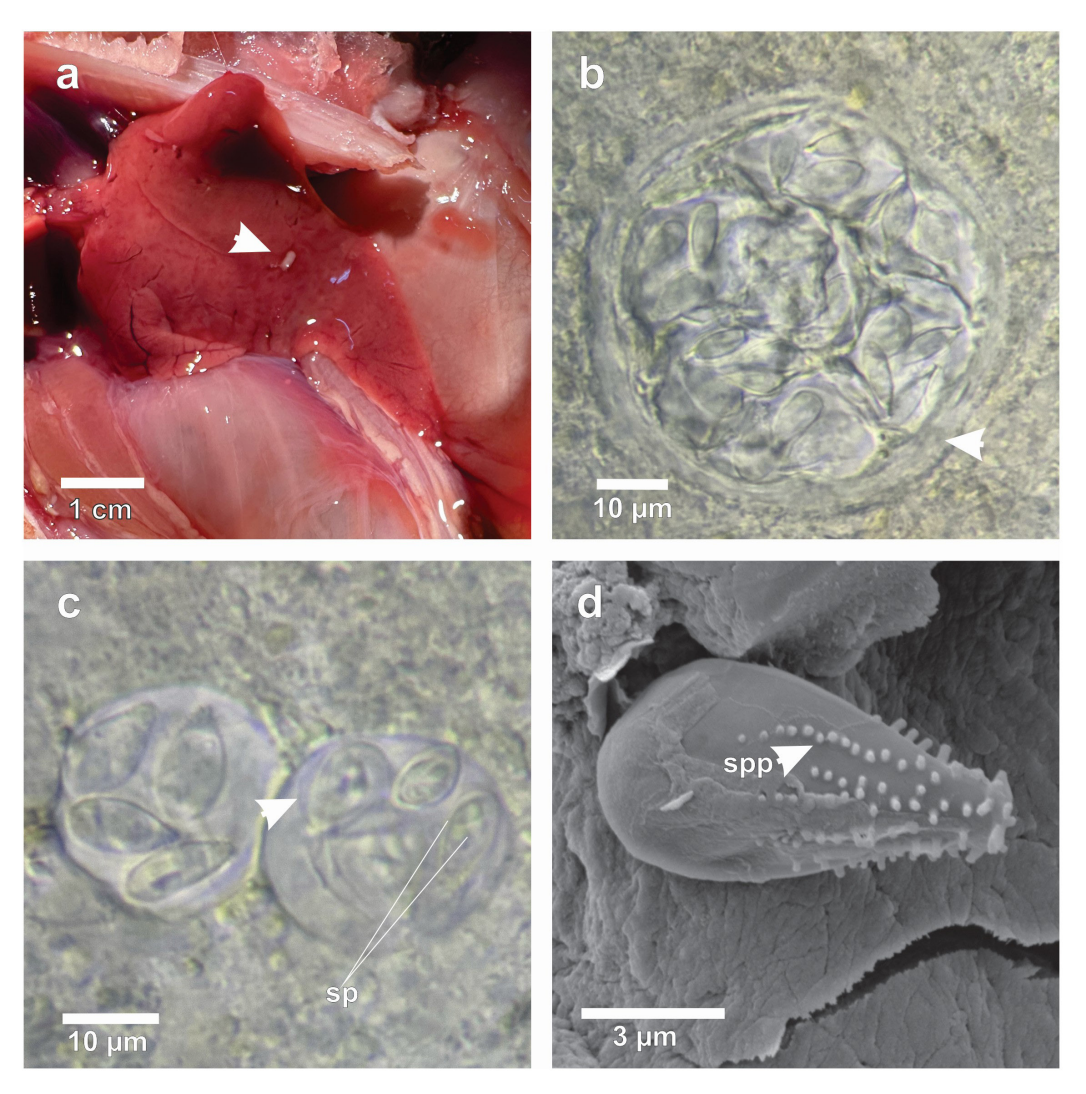
Macro and microphotographs of *Calyptospora* sp. infection in *Pygocentrus nattereri*. (A) Liver showing a whitish region with a cystic appearance. (B) Clusters of oocysts surrounded by a thick wall with a fibrous appearance observed under light microscopy. (C) Oocysts containing 4 sporocysts, sporozoites (sp) inside and the membranous veil (arrowhead). (D) Scanning electron microscopy micrographs of a sporocyst of *Calyptospora* sp. with emphasis on sporopods (spp).

Among the 18 necropsied *P. nattereri* specimens, six presented whitish regions in the liver (Figure 1a), with the appearance of clusters distributed throughout the liver parenchyma. Optical microscopy confirmed that the clusters were coccidial oocysts, present in large numbers, forming a thin wall of connective tissue around them and distributed throughout the liver parenchyma (Figure 1b); Isolated mature spherical oocysts were also observed and measured 18.1 ± 0.5 (17.4-18.7) µm in diameter, with four pyriform sporocysts measuring 9.0 ± 0.45 (8.3-9.8) µm in length and 4.3 ± 0.50 (3.7-5.1) µm in width, and it was possible to observe the membranous veil surrounding the sporozoites (Figure 1c). SEM revealed numerous sporopods concentrated on the surface of the narrow posterior end of the sporocyst (Figure 1d).

Due to the spherical shape of the oocysts containing four pyriform sporocysts, each with two sporozoites, these were identified as oocysts of an unidentified species in the genus *Calyptospora*, with a prevalence of 33.3%.

**Figure 2:**
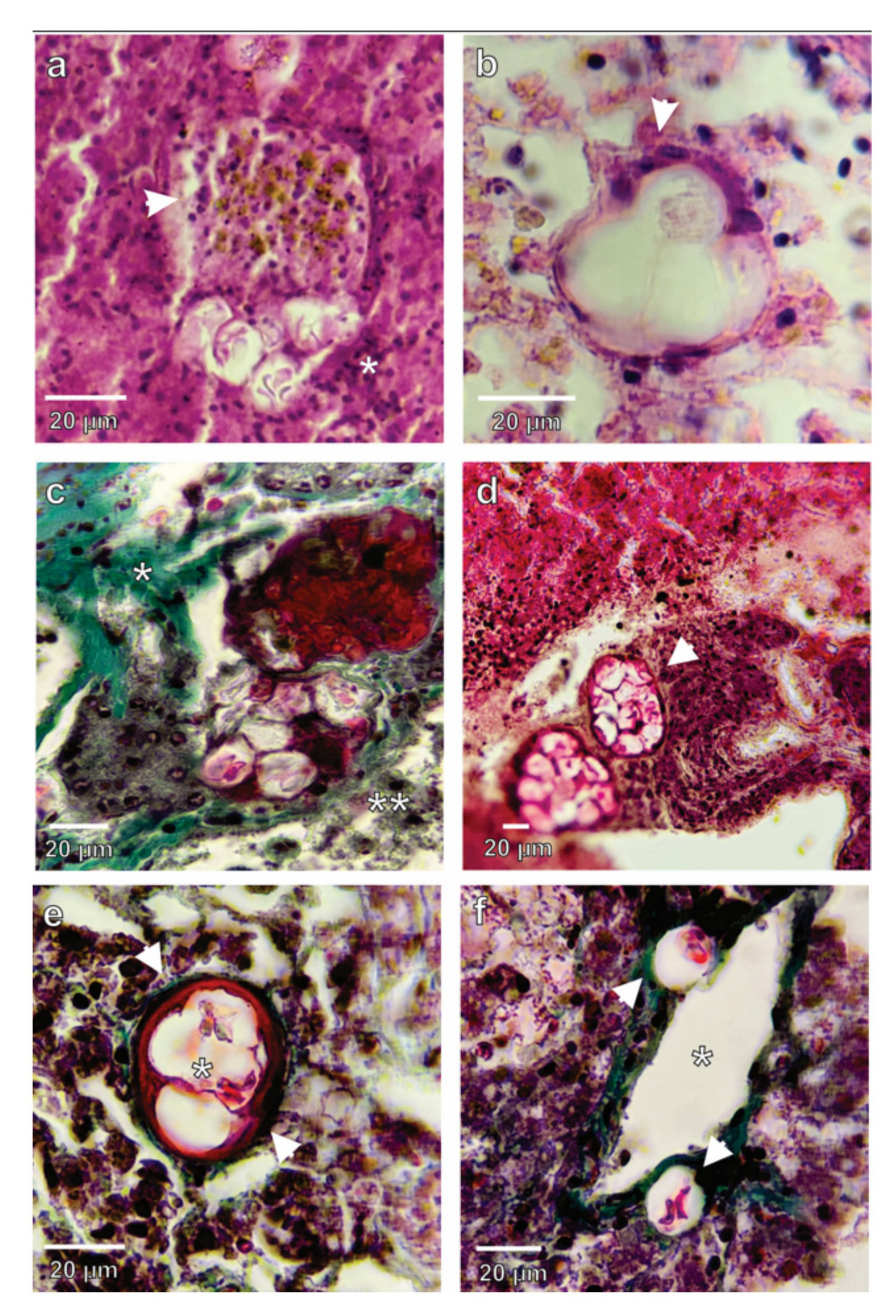
Microphotographs of histological sections of liver tissue from *Pygocentrus nattereri* infected by *Calyptospora* sp. A and B – Histological liver sections stained with HE. (A) Grouped oocysts compressing the hepatocytes and the presence of melanomacrophagic cells (arrowhead) full of yellowish pigment granules and inflammatory infiltrate (*). (B) Clusters of oocysts surrounded by a wall of fibroblasts (arrowhead). C, D, E, and F – Gomori trichrome staining: (C) Parasites of the genus *Calyptospora* causing fibrosis. Green coloring indicates the presence of collagen fibers (*) and degeneration (**) in the liver parenchyma and intrahepatic pancreas. (D) Oocysts clustered in the hepatopancreas region. (E) Cluster of oocysts in the intrahepatic pancreas region surrounded by a thick, fibrous wall (arrowhead). (F) Oocysts close to the blood vessel of the liver parenchyma (*).

Histopathological findings showed grouped oocysts compressing the hepatocytes and cells of the intrahepatic pancreas, along with melanomacrophage bodies containing pigment granules with a yellowish-brown color and inflammatory infiltrate (Figure 2a). The oocyst clusters were surrounded by a connective tissue wall with fibroblasts that clearly defined a single cystic layer (Figure 2b). We also observed tissue degeneration and compressive necrosis (Figure 2c and 2d), with liver cells exhibiting pyknotic and irregular nuclei and a lacy cytoplasm, particularly notable were emphasizing oocysts in the vicinity of blood vessels (Figure 2e).

Records exist of *Calyptospora* species parasitizing teleost fish from the families Pimelodidae, Cichlidae, Arapaimidae, Triportheidae, and Serrasalmidae, infecting the livers of these hosts and causing histopathological changes in the tissues (Silva et al., 2020; Oliveira et al., 2021). In the present study, *Calyptospora* sp. infection was recorded in the liver of *P. nattereri* with a prevalence of infection similar to that recorded in *Serrasalmus rhombeus* Linnaeus, 1766 (33.0%). However, *Serrasalmus striolatus* (Steindachner, 1908) higher prevalence values of *Calyptospora* at 53.3%, followed by *Cichla temensis* Humboldt, 1821, at 56%, *Brachyplatystoma vaillantii* (Valenciennes, 1840) at 60%, *Crenicichla lepidota* Heckel, 1840, at 63% and S*. rhombeus*, at 80%.

Currently, there are no records of *Calyptospora* infection in *P. nattereri*. However, such infections have been reported in other species of the Serrasalmidae family, including *Calyptospora serrasalmi* parasitizing *S. striolatus* and *S. rhombeus* (Casal et al., 2007)*.* The morphology of the oocysts and the arrangement of the sporopods in the sporocysts of *Calyptospora* sp. were similar to those of *C. serrasalmi* described by Whipps et al. (2012).

Studies on parasitism by *Calyptospora* have frequently reported the presence of whitish regions in the livers of host fish (Azevedo et al., 1993; Velasco et al., 2012; Videira et al., 2013; Oliveira et al., 2021). Furthermore, *Calyptospora* can damage the liver of fish at the cellular level, causing nuclear hypertrophy, destruction of the cytoplasm, and even severe degeneration of the organ (Békési & Molnár, 1991; Azevedo et al., 1993), which can lead to host death due to liver failure (Bonar et al., 2006). In the present study of *P. nattereri*, the presence of oocysts in the vicinity of blood vessels, fibrosis around clusters of oocysts in the hepatopancreas, tissue degeneration, and compressive necrosis were recorded, as reported in *S. rhombeus* by Oliveira et al. (2021).

The presence of inflammatory infiltrates and melanomacrophagic cells near the clusters of oocysts found in the liver of *P. nattereri* demonstrates the establishment of an infection process and the host's immunological response to parasitism (Bonar et al., 2006). These changes caused by *Calyptospora* have been recorded in the livers of *Cichla ocellaris* Bloch & Scheneider, 1801 (Békési & Molnár, 1991), *C. temensis* (Velasco et al., 2012), *Aequidens plagiozonatus* Kullander, 1984 (Videira et al., 2013), *Cichla piquiti* Kullander & Ferreira, 2006 (Silva et al., 2019) and *S. rhombeus* (Oliveira et al., 2021). However, there are cases in which, despite the occurrence of histopathological changes caused by *Calyptospora* in the hepatopancreas of the host no serious inflammatory processes have been observed in the tissue, such as those described in *Arapaima gigas* Cuvier, 1829 (Bonar et al., 2006) and *C. temensis* (Santiago et al., 2012).

This is the first documented case of coccidiosis caused by *Calyptospora* sp. in *P. nattereri* from Pracuúba municipality, Amapá state. These infections result in significant liver disease. Oocysts form fibrous cysts, that trigger recruitment of inflammatory cells and melanomacrophage bodies to the site of infection. Consequently, the liver parenchyma undergoes degeneration. These findings demonstrate that infection with *Calyptospora* parasites can cause liver damage and potentially affect the overall physiology of the host.
